# ‘Choice, culture and confidence’: key findings from the 2012 having a baby in Queensland Aboriginal and Torres Strait Islander survey

**DOI:** 10.1186/1472-6963-14-196

**Published:** 2014-05-01

**Authors:** Susan Parker, Loretta McKinnon, Sue Kruske

**Affiliations:** 1 Queensland Centre for Mothers & Babies, The University of Queensland, Brisbane, Australia

**Keywords:** Birthing on country, Indigenous, Aboriginal, Torres Strait Islander, Maternity care

## Abstract

**Background:**

To describe the maternity care experiences of Aboriginal and/or Torres Strait Islander women in Queensland, Australia and to identify areas for policy and practice improvements.

**Methods:**

A culturally-tailored survey requesting both quantitative and qualitative information was completed by respondents either independently (online or in hard copy) or with the assistance of a trained peer-interviewer. Data were analysed using descriptive statistics and thematic analysis.

Eligible women were over 16 years of age, identified as Aboriginal and/or Torres Strait Islander, resided in Queensland, and had a live, singleton birth between the first of July 2011 and the first of July 2012.

**Results:**

187 women of 207 respondents were included in analyses. Women reported high rates of stressful life events in pregnancy, low levels of choice in place of birth and model of care and limited options to carry out cultural practices. High levels of confidence in parenting were also reported. Women were less likely to report being treated with kindness, understanding and respect by maternity care staff than women answering a similar mainstream survey.

**Conclusions:**

Aboriginal and Torres Strait Islander women have additional needs to mainstream Australian women. This study identified a number of recommendations to improve services including the need to enhance the cultural competence of maternity services; increase access to continuity of midwifery care models, facilitate more choices in care, work with the strengths of Aboriginal and Torres Strait Islander women, families and communities, and engage women in the design and delivery of care.

## Background

Aboriginal and/or Torres Strait Islander^1^ women have a higher prevalence of factors associated with negative health outcomes for mothers and newborns compared to their non-Indigenous counterparts [1-5]. Aboriginal and/or Torres Strait Islander women are also at risk of not receiving culturally tailored care during the maternity period [6-8] and have frequently reported dissatisfaction with hospital-based maternity care experiences [9-11]. Birthing is often considered a fearful time for many women, regardless of ethnicity [12]. For Aboriginal and/or Torres Strait Islander women this experience can be even more difficult because of the lack of culturally competent mainstream systems [7], previous negative experiences with the health care system, and a high burden of disease [13]. In addition, higher proportions of Aboriginal and/or Torres Strait Islander women are required to leave their communities and relocate for birth resulting in birthing without support people, worrying about their children left behind and suffering logistical and financial burdens related to travelling [14,15]. Aboriginal and/or Torres Strait Islander women are often subject to a broad range of life stressors such premature and unexpected death of family members, financial stress, family violence and socio-economic disadvantage [5,16-18]. Finally, many Aboriginal and/or Torres Strait Islander women and families continue to suffer the impact of racism, colonisation and the stolen generation, which can result in fear and distrust of government services and the hospital environments [7].

Many of the directions of current Queensland [19] and National [20] policy documents support improvements in maternity care available to Australian women with specific attention given to Aboriginal and/or Torres Strait Islander women. In particular, these reforms aim to enhance consumer involvement and choice in relation to maternity care and to ensure that quality, evidence-based, safe and culturally competent care is delivered by an appropriately trained workforce within a sustainable maternity care system [19,20].

Obtaining consumer input is critical for the design, implementation, and evaluation of maternity care services [21]. Published work around the maternity experiences of Aboriginal and Torres Strait Islander women in Queensland is limited [22,23]. This study aimed to explore the maternity care experience of Aboriginal and/or Torres Strait Islander women birthing in Queensland using mixed methods and peer interviewer techniques previously not utilised in this area of research. Both quantitative and qualitative data were assessed to determine opportunities for improving the delivery of maternity care services to this population.

## Methods

### Research design

The research design involved the construction of a culturally tailored data collection instrument and selection of sample recruitment and data collection methods designed to maximise representation and access to Aboriginal and/or Torres Strait Islander women.

### Geographic sample selection

A stratified, purposeful sampling technique was used to ensure a diverse geographical sample of participants. Six areas across Queensland were chosen for their high number of Aboriginal and/or Torres Strait Islander births and to reflect a mix of urban, regional and remote dwelling women. These were: Brisbane; Townsville/Palm Island; Mount Isa (including Doomadgee and Mornington Island); Thursday Island; Rockhampton; and Woorabinda.

### Data collection instrument

The survey [24] was adapted from the mainstream 2012 Having a Baby in Queensland (HABIQ) survey [25] to improve cultural relevance to Aboriginal and/or Torres Strait Islander women’s maternity care experience. Modifications from the mainstream survey included the changing of wording to improve readability and meaning for an Aboriginal and or Torres Strait Islander audience. Additional items were added to capture experiences known to impact on Aboriginal and Torres Strait Islander women. These included stressful events in pregnancy, specific cultural practices and experiencing racism through items that elicited feeling judged, being talked down to and being treated as an individual.

The 24 page survey was adjusted based on a pilot study that tested the suitability and readability of the tool with five Aboriginal women who had recently had a baby outside of Queensland and were known to the Aboriginal researcher on the team. Women were asked about the quality of care received during pregnancy, labour and birth and postnatal periods and were invited to provide comments on suggested improvements to care. Demographic characteristics were also collected.

Some survey items required respondents to select from a number of pre-determined responses (including likert scales). For example, respondents were asked the question “Where was your baby born?” with response options including ‘public hospital’, ‘private hospital’, ‘birth centre’, ‘at home’, ‘not sure’ or ‘other’. Data derived from these questions are described through this paper as ‘quantitative’ data. Other questions in the survey requested an open-ended response, where respondents could provide what they felt was the relevant detail without any limitation with regard to the topics they could raise. An example of such an open-text question was “What were the good things about your pregnancy care?”. Throughout this paper, data collected from these type of open-ended questions are described as ‘qualitative’ data.

### Sample selection and recruitment

Aboriginal and/or Torres Strait Islander women who had a live singleton birth in Queensland between I July 2011 and 1 July 2012 were invited to participate. The age requirement was 16 to ensure that respondents were of an age where they could consent and so that young mothers were not excluded from participating. Consistent with other studies investigating the health experiences of Aboriginal and/or Torres Strait Islander populations [26], multiple methods were used to recruit participants. The study was promoted via social media (including facebook and twitter), through posters in, maternity services, community hubs (e.g. Aboriginal Community Controlled Health Organisations) and using stalls at local events (e.g. National Aboriginal and Islander Day of Commemoration (NAIDOC). Consent to contact forms were available at the places for women to indicate their interest in participating.

Peer interviewers were recruited to administer the survey across the six designated project sites and were supported by the Aboriginal project lead (first author). They were local Aboriginal and/or Torres Strait Islander women well-connected to their community with good verbal and written communication skills. Use of peer interviewers was to enhance the engagement of a broad range of Aboriginal and/or Torres Strait Islander women and to facilitate interviews being conducted in a sensitive and culturally appropriate manner (thus enhancing the quality of the data collected). In total, 28 peer interviewers (age range: 20 to 60) were recruited across the project sites. To allow greater flexibility and reduced monitoring and recording of hours worked, peer interviewers were paid per survey completed, rather than per hour.

Training was provided to all of peer interviewers to ensure that they understood the project objectives, their role in the project, and how to follow research procedures such as gaining consent, data collection and storage. The training also focused on the development of practical skills such as how to recruit participants to do the survey, learning interview skills and what action to take when women needed to access further support from appropriate health professionals. Peer interviewers were actively involved in promoting the survey, and recruiting participants from their own informal networks.

### Data collection and analysis

Data collection occurred between July and October 2012. Women could complete the survey either online or using a hard copy, or by either method with the assistance of a peer-interviewer. Data collected via peer interviewers varied with most interviews conducted at the participant’s home or in cafes. A total of 169 (90%) women used peer interviewers to complete the survey and 18 (10%) completed the survey individually. A number of peer interviewers had their laptops and internet connection and were able to enter the information directly while they conducted the survey. Only two peer interviewers completed surveys in this way with the remainder using hard copy survey forms. Thirty one surveys were completed on line (including those by peer interviewers) with the remainder being completed on hardcopy.

The quantitative analysis involved the generation of descriptive statistics using SPSS (version 21). The qualitative data were subject to a thematic analysis undertaken by the first and second authors. NVivo (version 9) was used for these analyses. Data familiarisation was undertaken whereby the researchers read and re-read the data to become familiar with the content. The data were then systematically assessed for the identification of ideas, meanings, concepts and keywords and relevant ‘codes’ were developed. These codes were then compared and contrasted, and similar concepts were grouped into themes.

Each researcher independently assessed a number of open-text survey questions. Formal double-coding was not undertaken. However, the authors regularly met and discussed the decisions being made with regard to the coding of data and the development of themes. This collaborative analysis is likely to have enhanced the trustworthiness of the findings. Illustrative quotes are provided in the results section to further support the rigour of the analysis and the authenticity of the themes derived.

### Ethics

The study was approved by the Human Research Ethics Committee of the School of Psychology, University of Queensland. Written consent was obtained by peer interviewers following reading out the information sheet and prior to completing the survey. Consent was obtained from women filling out the survey on line through the process of clicking on the ‘I Consent’ button at the bottom of the information statement prior to progressing to the survey instrument.

## Results

### Participant characteristics

Demographic characteristics of the women are presented in Table One. A total of 187 women of 207 respondents were included in analyses. This represents approximately 5% of the 3649 Aboriginal and/or Torres Strait Islander women who birthed in Queensland in 2011 [27].

The sample was representative of Aboriginal and/or Torres Strait Islander women in Australia in terms of location, parity and age characteristics [28]. A higher proportion of women in the sample identified of being of Torres Strait Islander descent (9.6%) or of both Aboriginal and/or Torres Strait Islander decent (19.3%) compared to the total Australian population (respectively 6% and 4%) [29]. The sample was more educated that the overall Indigenous population with 10.2% of respondents indicating that they had completed tertiary studies, compared to the national average of 5.0% for Indigenous Australians [30]. This observation is consistent with known research participation patterns wherein generally higher proportions of educated or affluent persons participate in research compared to those who are less advantaged [31] Table 1.

**Table 1:** Participant characteristics (n = 187)

	**%**	**(n)**
**Indigenous status (self-reported)**		
Aboriginal	71.1	(133)
Torres Strait Islander (TSI)	9.6	(18)
Both Aboriginal and TSI	19.3	(36)
Age (at time of birth)		
16-24	52.9	(99)
25-29	23.0	(43)
30-39	23.0	(43)
>40	1.1	(2)
**Education (highest level completed)**		
Did not complete primary school	0.5	(1)
Primary school	5.3	(10)
Year 10 or equivalent	44.9	(84)
Year 12 or equivalent	34.8	(65)
Trade/apprenticeship	4.3	(8)
Tertiary qualifications	10.2	(19)
**Area of residence**^**b**^		
Major cities	34	(64)
Outer regional	44	(83)
Remote and very remote	20	(38)
**Have other children**^**a**^		
Yes	63.1	(118)
No	35.8	(67)

^a:^ This description is used rather than parity as women were not asked about the number of times they had previously given birth, rather they were asked whether or not they had other children.

^b:^ based on the Accessibility and Remoteness Index of Australia.

### Antenatal service delivery

The survey investigated a number of aspects of antenatal care including the timing, number and experience of antenatal appointments.

#### Timing of first antenatal visit

Survey respondents first saw a health professional regarding their pregnancy at an average gestation of 7.9 weeks (SD 3.71 weeks; range 2 to 28 weeks).

#### Number of check-ups while pregnant

A minimum number of five antenatal appointments is a national performance indicator [2] due to its association with improved outcomes [32]. Approximately two thirds of women (*n* 126, 67.4%) attended at least five antenatal appointments. The remaining women attended either one (*n* 2, 1.0%), two (*n* 8*,* 4.3%), three (*n* 14, 7.5%), or four (*n* 19, 10.2%) appointments, or did not provide data for this question (*n* 18, 9.6%).

#### Choice of gender of health care provider

Women were asked whether they could choose between male or female care providers during pregnancy. The response options were ‘Yes’, ‘No’, and ‘Unsure’. Over one third of women responded that they could not choose their care provider’s gender (*n* 69, 36.9%) or were unsure whether a choice was available (*n* 64, 34.2%). The remaining women indicated that they could choose the gender of their care provider (*n* 52, 27.8%).

#### Acceptability of questions about particular health topics during the antenatal period

**Figure 1:**
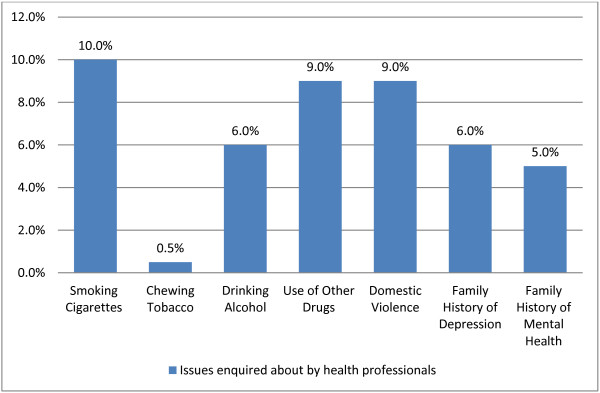
Proportion of women who felt uncomfortable being asked about various topics.

Women were asked how comfortable they felt being asked routine questions on a range of issues by their health professional. Approximately ten percent of women (*n* 19, 10.2%) indicated that they felt uncomfortable when asked about various issues by health professionals during their pregnancy. Among the topics that made women feel uncomfortable, smoking cigarettes, use of other drugs, and domestic violence were the most commonly mentioned topics (see Figure 1).

#### Stressful life events during pregnancy

**Figure 2:**
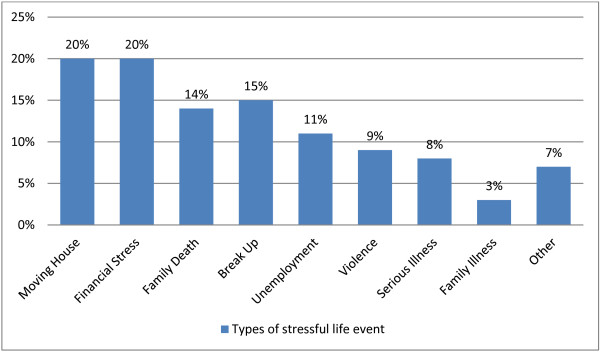
Proportion of women experiencing various stressful life events during pregnancy.

The majority of women indicated that they had experienced at least one stressful life event during their pregnancy (*n* 116, 62%). Such stressful life events included moving house, financial stress or a death in the family. The proportion of the sample experiencing each type of stressful life event is shown in Figure 2.

### Birthing and hospital stay

The majority of women (*n* 183, 97.3%) birthed in a public hospital, with the remainder of women birthing either in a birth centre (*n* 3, 1.6%) or at home (*n* 1, 0.5%). Two thirds of women (*n* 123, 66%) indicated that they did not have a choice as to where they could have their baby. Two thirds of women indicated that an Indigenous specific service was available to them however, only half of women with access to such a service chose to use it.

Women were asked *“Were you able to carry out any cultural practices during your pregnancy and birth?”*. Very few women (*n* 24, 12.8%) indicated that they were able to carry out any cultural practices during their pregnancy and birth. A number of women made comments to indicate that they did not realise that engaging in cultural practices within the hospital setting was an option available to them since an invitation to participate in such practices was not made by hospital staff.

“I didn’t ask, I just presumed that I would not be able to do this in the hospital” (Respondent 48, Inner regional, had other children)

Some, but not all, maternity staff, were perceived as being supportive of women engaging in cultural practices surrounding birth.

“If I wanted to I don't think there would have been an issue” (Respondent 79, Major city, had other children)

“Doctors told me I could not take my placenta out of surgical room due to contamination” (Respondent 144, Major city, had other children)

### Post natal experiences

Women were asked to report how confident they felt in looking after their baby once they had returned home. Women’s responses were: ‘extremely confident’ (*n* 97, 52%), ‘fairly confident’ (*n* 47, 25%), ‘confident’ (*n* 26, 14%), ‘not very confident’ (*n* 9, 5%), ‘my baby hasn’t come yet’ (*n* 2, 1%) and missing data (*n* 4, 2%). When parity was considered, as expected, multiparous (who reported having other children) women were more confident caring for their baby when they returned home. Specifically, 63.6% (*n* 75) of multiparous women reported being ‘extremely confident’ compared to 35.8% (*n* 24) of women having their first baby.

Approximately a quarter of the women interviewed indicated that they did not feel that they had anyone to talk to with regards to how they were feeling after the birth of their baby (*n* 50, 27%). An equally low number of women (*n* 33, 18%) indicated that they had joined a mothers and babies group in their local area.

### Maternity care service delivery overall

#### Continuity of carer during pregnancy

The majority of women (*n* 136, 72.7%) indicated that they saw the same health professional during their pregnancy. Throughout the survey, when women were asked to articulate the positive aspects of their care, women commented on their satisfaction with having a known midwife or the same midwife during their pregnancy. The quote below is exemplary regarding the type of comments women made about the way that care from a known provider enhanced their maternity care experience.

“Having the same midwife & her personal contact details to call anytime” (Respondent 8, Major city, had other children).

#### Relocating for birth

Just under one third of women left their community in order to give birth (*n* 53, 28.3%). All lived in an outer regional, remote, or very remote community. Almost all of these women had a support person (*n* 45, 85%) however, 15.1% (*n* 8) did not. Most of those who travelled with a support person (*n* 31, 69.0%), indicated that their support person did not receive any financial assistance to accompany them when travelling to give birth.

Among women who travelled for birth the majority of women (*n* 32, 60.0%) indicated that they would have preferred to stay in their own community to give birth. These women described issues associated with childcare, interruption to their partner’s work in order to travel, feelings of isolation from not having partner or family there with them and a lack of adequate financial assistance for travel and accommodation. Below are some illustrative quotes from women who indicated that they would have preferred to stay in their community to give birth.

“It would have saved a lot of hassle and money” (Respondent 126, Remote location, had other children)

“It would have been a lot easier as my eldest is school age & it's hard to go away for a whole month” (Respondent 7, Inner regional, had other children)

#### Interpersonal aspects of care

Women were asked about a number of interpersonal aspects of their care during pregnancy, labour, birth and during the postnatal period. Specifically, women were asked how often they were: treated as an individual; treated with kindness and understanding: not ‘talked down to’; and not ‘ignored’ by health staff. The response options were ‘always’, ‘often’, ‘sometimes’, ‘rarely’, ‘never’, or ‘prefer not to say’. The proportions of women who indicated how often they were treated in each of the manners addressed are presented in Table 2 with the most popular response for each question shown shaded. As can be seen in Table 2, just over half of the women (more than 59%) indicated that throughout their care they were always treated with respect, as an individual or with kindness and understanding.

**Table 2:** Women’s evaluation of five components of communication with hospital staff during three time periods*

**A**	**During your labour and birth did you feel like the health professionals or other staff:**									
	**Treated you with respect**		**Treated you as an individual**		**Treated you with kindness and understanding**		**Talked down to you**		**Judged, insulted or ignored you**	
	**%**	**(n)**	**%**	**(n)**	**%**	**(n)**	**%**	**(n)**	**%**	**(n)**
Always	68	(128)	61	(114)	64	(119)	3	(6)	3	(5)
Often	16	(29)	15	(28)	17	(32)	3	(6)	5	(9)
Sometimes	10	(19)	11	(20)	13	(24)	10	(18)	8	(15)
Rarely	1	(2)	3	(6)	2	(3)	9	(16)	5	(10)
Never	2	(3)	5	(10)	2	(3)	71	(132)	75	(140)
**B**	**After giving birth while you were still in hospital did you feel like the health professionals or other staff:**									
	**Treated you with respect**		**Treated you as an individual**		**Treated you with kindness and understanding**		**Talked down to you**		**Judged, insulted or ignored you**	
	**%**	**(n)**	**%**	**(n)**	**%**	**(n)**	**%**	**(n)**	**%**	**(n)**
Always	68	(128)	59	(111)	69	(129)	3	(5)	2	(3)
Often	14	(26)	15	(28)	15	(28)	2	(4)	2	(4)
Sometimes	10	(19)	13	(24)	10	(19)	9	(16)	9	(16)
Rarely	2	(3)	2	(3)	1	(2)	9	(17)	9	(17)
Never	0	(0)	5	(10)	0.5	(1)	70	(131)	71	(133)
**C**	**After giving birth when you returned home did you feel like the health professionals or other staff:**									
	**Treated you with respect**		**Treated you as an individual**		**Treated you with kindness and understanding**		**Talked down to you**		**Judged, insulted or ignored you**	
	**%**	**(n)**	**%**	**(n)**	**%**	**(n)**	**%**	**(n)**	**%**	**(n)**
Always	68	(128)	61	(114)	70	(130)	(3)	5	3	(6)
Often	18	(33)	18	(33)	19	(36)	(3)	5	2	(4)
Sometimes	6	(11)	6	(12)	2	(4)	(3)	5	2	(3)
Rarely	1	(2)	2	(3)	1	(2)	(12)	22	8	(15)
Never	0.5	(1)	6	(12)	2	(3)	(72)	136	78	(145)

The qualitative analysis yielded further information on what women thought were the ‘good’ and ‘not so good’ aspects of their care during pregnancy. Women described ‘good’ aspects of their care as seeing the same person, or a familiar person throughout their care.

“Having the same midwife help guide me through each stages of my pregnancy. The midwife was with me also during my doctors’ appointments which was a big help, as they knew the right questions to ask…” Respondent 110, urban location, primparous.

The comments of women also reflected how the expression of empathy and kindness by caregivers enhanced their experience. The ‘not so good things’ reported by women included long wait times, not being able to be seen by the same doctor or midwife, rude staff and costs associated with travelling and maternity care.

“Waiting for ages in the waiting room. One time I waited for an hr and a half because the midwife took the wrong slip.” Respondent 165, remote location, primiparous.

#### Transport

**Figure 3:**
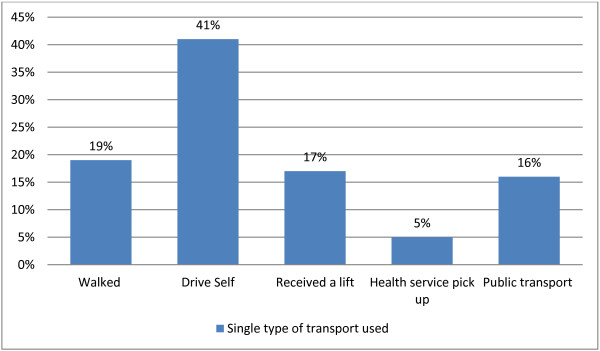
Distribution of the types of transport used to attend appointments during pregnancy.

Women were asked what mode of travel they used to attend most of their pregnancy check-ups. The response options were: ‘walked’; ‘drove own car’; ‘got lift from partner, friends or family’; ‘health care worker picked me up’; ‘public transport (e.g. bus, train)’; or ‘other’. Women could report using as many modes of transport as were applicable, however most women reported using a single mode of transport (*n* 150, 80%). The type of transport used by these women who used a single mode are shown in Figure 3. Among those who used multiple forms of transport (*n* 32, 16.6%), most got a lift from a partner friend or family member (*n* 24, 72.7%) in addition to walking, driving themselves or public transport.

## Discussion

This study explored Aboriginal and/or Torres Strait Islander women’s perceptions of care during pregnancy, labour, birth and the postnatal period. Aboriginal and/or Torres Strait Islander women’s satisfaction with maternity care has been measured in a number of previous studies [22,23,33-35].

### Antenatal care

The average time at which women first saw a health professional in their pregnancy was 7.9 weeks (SD 3.71; range 2 to 28 weeks). Whilst this represents an earlier timeframe outlined in a recent paper which found the average time of first prenatal visit for Aboriginal women in South Australia as 10.1 weeks (SD 4.8) [26]. It is, however, similar to the average time of first visit among all women in the HABIQ 2012 sample [25] that reported an average of 8.0 weeks gestation (SD 3.90; range 0 to 32 weeks).

Increasing access to quality antenatal care for Aboriginal and/or Torres Strait Islander women has been noted as an important goal by a number of authors [22,36,37] and changes to antenatal care delivery has been found to increase service use by this population in some parts of Queensland [37]. Three factors associated with increased participation in antenatal care identified by Ship et al. include: awareness of community services, attendance at childbirth education classes and interest from father and extended family [38].

### Postnatal care

The participants in this survey indicated a lack of postnatal support with high proportions of women (63%) indicating that they had no-one to talk to about how they were feeling after the birth of their baby and/or who had not joined a local mothers group (76%). These findings support other calls to ensure Aboriginal and/or Torres Strait Islander women have access to quality care and support during pregnancy, birth and the postnatal period [5,39]. Significant investment into strengthening Indigenous maternal and child health services has occurred across Australia in recent years through programs such as the New Directions: Mothers and Babies Services [40], Healthy for Life [41] and Australian Nurse Family Partnership [42]. Effectiveness of these programs on Aboriginal and Torres Strait Islander women and babies outcomes is not well captured [40].

### Feeling respected

Women were asked about a number of interpersonal aspects of their care during pregnancy, labour, birth and during the postnatal period. Over half of the women (≤59%) expressed satisfaction with care, with approximately 30% indicating that they were not ‘always’ treated as an individual, treated with kindness and understanding, not ‘talked down to’, and not ignored, by medical staff. Woman-centred care is a current national maternity reform objective [20] and has been defined as:

*‘a process in which a woman makes choices and is involved in and has control over her care…’*[43].

The results of this study imply that for a considerable number of women a central tenet of women-centred care, being respected, may not be being met.

Dietsch and colleagues [44] reported that Aboriginal women were being more likely to be treated poorly by maternity staff, describing experiences that were interpreted as bullying. Approximately 70% in our study reported ‘always’ being treated with respect or not being talked down to. This raises issues for the remaining 30% women who indicated an alternative experience.

Differences for Aboriginal and/or Torres Strait Islander women compared to mainstream women can be found in the higher proportions of women in the mainstream HABIQ 2012 study [25], who reported ‘always’ being treated with respect (76.7% vs 68%), treated as an individual (75.4% vs 61%), and treated with kindness and understanding (75.7% vs 64%) [25].

### Choices in maternity care

The lack of choices available to Aboriginal and/or Torres Strait Islander women has been documented in a number of publications and reports [15,45-48]. The type of choices advocated for include the geographical location of birth [48], model of care received [33,36], choice of who is present at the birth and whether culturally important protocols are adhered to [15]. This study collected data relevant to maternity care choices including whether women had a choice of male or female care providers during their pregnancy, whether women would have preferred to stay in their local community to give birth, the place of birth, and whether continuity of care was available.

Over two thirds of women (71.1%) indicated that they were either not able, or were unsure whether they were able, to choose the gender of their care provider during pregnancy. Many Aboriginal and/or Torres Strait Islander women have cultural beliefs regarding gender roles and participation during the maternity period and have reported feelings of ‘shame’ in being cared for in birth by men [15] and when discussing reproductive health issues in general [49]. Therefore, not having a choice in the gender of caregivers could cause distress for some Aboriginal and/or Torres Strait Islander women.

Sixty percent of women who relocated to give birth indicated that, given the choice, they would have stayed in their local community to give birth. Results also suggest that women may have had a limited choice of birthing venues since the majority of women surveyed birthed in public hospitals (97.3%), with very few women birthing in birth centres (1.6%) or at home (0.5%). This assertion that there was a lack of choice in venue was supported by the majority of women (66%) expressing that they did not have a choice in terms of where they gave birth.

A number of authors indicate that choice is limited for many Aboriginal and/or Torres Strait Islander women with the focus of existing maternity care delivery being on bio-physical safety, with the provision of culturally safe care being a lower priority [6,15,48]. The provision of choices in maternity care for all Australian women is a goal of Queensland specific and national maternity care reforms [19,20] and should be prioritised for this population.

### Cultural practices in childbirth

Only a small proportion of women indicated that they were able to carry out any cultural practices during their pregnancy and birth (12.8%). When asked whether they carried out any cultural practices a number of women mentioned that they were not invited to carry out any cultural practices by maternity care staff. This may suggest that without being asked, Aboriginal and/or Torres Strait Islander women may have felt that they could not engage in cultural practices.

### Travelling for birth

Other studies [11,35] have reported women’s’ feelings of loneliness and fear, due to being sent away from their own communities for birth. These findings were also supported by women in this current study who indicated that travelling away for birth caused additional stress. Travelling in order to give birth has been reported as being both socially and emotionally taxing for Aboriginal and/or Torres Strait Islander women [15,33,48]. Poverty, and or inadequate financial resources has been noted as an additional stressor for Indigenous women travelling to give birth both in Canada [48,50] and Australia [17,51]. Aboriginal and/or Torres Strait Islander women relocating for birth incur expenses including, their own travel and accommodation costs and those of their support person [22,52]. They may also incur childcare costs for children that are left at home. This study particularly highlighted the inadequacy of financial subsidies, and resulting stress for women who were required to travel to give birth whom comprised nearly one third of the women sampled (28.3%).

### Recommendations for policy and practice

A number of opportunities to improve women’s experiences of care were identified. These include the need to: enhance the cultural competence of maternity services; improve access to continuity of midwifery care models; facilitate more choices in care; and, work with the strengths of Aboriginal and Torres Strait Islander women, families and communities.

### Culturally competent care

Internationally it has been acknowledged that women’s perceptions and needs during childbirth are socio-culturally defined [53]. Several Australian publications have emphasised the limitations of maternity services in recognising this [7,15,33,54]. Cultural competence has been defined in terms of the characteristics of organisations and individuals as:

*“Organisations [that] have a defined set of values and principles, and demonstrate behaviours, attitudes, policies and structures that enable them to work effectively cross-culturally. Cultural competence is a developmental process that evolves over an extended period. Both individuals and organisations are at various levels of awareness, knowledge and skills along the cultural competence continuum”.*[55]

A lack of culturally appropriate care can result in shame for women and avoidance of the health system [15,33,37]. Providing culturally competent care is likely to improve health outcomes of Aboriginal and/or Torres Strait Islander women and their babies by increasing their use of maternity care services [56,57]. Care providers should be especially mindful of the high risk of this population to be subject to multiple life stressors including, commencing childbearing at a relatively young age [58] and being of low socioeconomic position [17,18]. The impact of many such known stressors for Aboriginal and/or Torres Strait Islander persons were demonstrated in the findings of this research, including moving house, financial stress, and exposure to violence [30,59]. Thus, this research reinforces the need for staff providing maternity care services to Aboriginal and/or Torres Strait Islander women to be aware of the additional life-stressors faced by this population that may impact on their health and coping during the maternity period.

Improving the education of the workforce specifically in terms of the provision of culturally competent care is highlighted as a state-wide [19] and national [20] reform objective. This study provides further support for the importance of these goals. Reports have been prepared specifically to assist with guiding the provision of culturally competent care to Aboriginal and/or Torres Strait Islander Australians and these should be consulted to optimally pursue this goal [7,22,60].

### Continuity of midwifery care

Aboriginal and/or Torres Strait Islander women have been reported positive experiences where care is received by a primary or small group of midwives, allowing women to develop trust through continuity of care [33,61]. While continuity of care across the full child care continuum (pregnancy, labour and post natal care) was not collected in this study, 72.7% indicated that they saw the same health professional during their pregnancy. Qualitative comments indicated that knowing their midwife was highly valued by women and supports the findings by Josif et al. [33].

Given that a high proportion of women (28.0%) travelled to give birth, continuity of care may need to be assessed differently for this group. In some Australian regions continuity of care from a known midwife is being successfully implemented for Aboriginal women living in remote areas [33,45,62] as well urban areas [34,63]. Recent Australian evidence suggests that midwifery-led continuity of care models are safe, cost effective [64] and are highly desired by women [33,65-67]. The opportunity for continuity of care is promoted as a national maternity reform objective as is providing choices of care to women in a range of settings, including rural settings where a disproportionately high number of Indigenous women are located [20].

### Working with strengths of women and community

This study found higher levels of confidence in parenting compared to Queensland women responding to the same questions in a mainstream survey in 2012 [22]. Irrespective of parity, a higher proportion of Aboriginal and/or Torres Strait Islander women reported the highest level of confidence (‘extremely confident’) in caring for their baby upon returning home (multiparous 63.6%, and primiparous 35.8%) compared to women in the 2012 HABIQ overall population study (multiparous 47.0%, and primiparous 10.6%) [25].

Although Aboriginal and/or Torres Strait Islander women experience greater risk factors [1-5] and suffer higher exposure to stressful life events [16-18] (which were supported by our findings), there are many strengths in these families that are often overlooked and underutilised by health service providers.

Confidence in parenting is one example of a strength that should not be overlooked. Family support, connection to country and cultural identify are other examples reported in the literature [68-73] but were not assessed in this current study.

While a strengths-based approach is promoted in the literature [74] this is often difficult to achieve given the different focus on risk between the health system and some women [6]. More effort by the maternity sector to work with women and community strengths should result in more engagement by women, higher compliance and better outcomes [69,73,75].

### Engaging women in the design and delivery of service

It is widely acknowledged that ensuring Aboriginal and/or Torres Strait Islander Australians governance and ownership regarding Indigenous maternity care delivery is likely to enhance maternity care satisfaction for this population [36,54,76]. Accessing women’s experiences is an important component of the engagement of women in the design and delivery of services. This survey demonstrates an effective strategy for achieving this. Additional strategies identified in the literature include: working with community elders and cultural knowledge brokers to ensure two way learning and knowledge in the delivery of care; the establishment of Aboriginal and/or Torres Strait Islander Advisory Groups; shared governance structures across mainstream and Indigenous agencies; family involvement and; the inclusion of Aboriginal and/or Torres Strait Islander personnel, especially female staff [45,77,78].

### Limitations and strengths of this study

The observational nature of this study does not allow conclusions about causality to be drawn from the data. The survey did not attempt to capture clinical data, nor did it measure a number of lifestyle factors known to contribute to poor perinatal health outcomes in Indigenous populations. These data are available elsewhere [79-81] and were not the focus of this study. Rather, the focus of this study was to investigate how maternity care was evaluated by Aboriginal and/or Torres Strait Islander women to inform improvements in service delivery. Although the responses represented only 5% of the Aboriginal and/or Torres Strait Islander births in 2011, it was representative in geographical location with good participation from remote and very remote dwelling women. Other strengths include the focus on women’s perceptions of maternity care services, the use of a culturally appropriate research design using peer interviewers and the application of mixed-methods.

Another unexpected limitation was the lack of qualitative information recorded by some interviewers. Remuneration for peer interviewers was per survey, rather than per hours worked. This was to increase flexibility for the peer interviewers and reduce the need for monitoring and recording of hours worked. This model could have been a disincentive for peer interviewers to encourage the women to talk more about their experiences, or record these conversations as qualitative data.

## Conclusion

This paper has provided some important insights into maternity care provision by accessing and reporting on the voices of birthing Aboriginal and/or Torres Strait Islander women in Queensland. Women reported high rates of stressful life events in pregnancy, low levels of choice in place of birth and model of care and limited options to carry out cultural practices. High levels of confidence in parenting were also reported. These findings indicate that Aboriginal and/or Torres Strait Islander women require specific and considered care that addresses their unique cultural, social and historical place in Australian society. Recommendations to improve women’s experiences of care include the need to: enhance the cultural competence of maternity services; improve access to continuity of midwifery care models; facilitate more choices in care; and, work with the strengths of Aboriginal and Torres Strait Islander women, families and communities.

## Competing interests

The authors declare that they have no competing interests.

## Authors’ contributions

SP designed the survey, analysed the data and revised the manuscript. L McK analysed the data and drafted the manuscript. SK conceived the study, contributed to the design and development of the manuscript. All authors read and approved the final manuscript.

## Pre-publication history

The pre-publication history for this paper can be accessed here:

http://www.biomedcentral.com/1472-6963/14/196/prepub
